# A wavelet-integrated framework for feature extraction and background refinement in hyperspectral anomaly detection

**DOI:** 10.1038/s41598-026-41223-w

**Published:** 2026-02-25

**Authors:** Fatma Küçük

**Affiliations:** Department of Software Engineering, Ankara Yıldırım Beyazıt University, Keçiören, 06010 Ankara Turkey

**Keywords:** Hyperspectral imaging, Anomaly detection, Wavelet transform, Mahalanobis distance, Engineering, Mathematics and computing, Optics and photonics

## Abstract

Wavelet transform (WT) has ability to analyze signals on different frequency bands with resolutions that vary to facilitate the detection of multiple aspects of a signal. With application of Haar WT on each pixel spectrum, the datasets are decomposed into low-frequency approximation coefficients representing essential spectral behaviour and high-frequency detail coefficients. It serves as a powerful preprocessing step for hyperspectral anomaly detection, as anomaly detection is becoming increasingly vital in hyperspectral image analysis. Traditional methods primarily rely on extracting background knowledge and distinguishing anomalies based on their difference from background, which often suffers from challenges such as anomaly contamination. This paper proposes a WT-based method for hyperspectral anomaly detection (WTHAD), which combines WT, Go Decomposition (GoDec) algorithm, and Mahalanobis Distance (MD). It fully exploits WT that offers a powerful preprocessing framework through the analysis of signals in different frequency bands with different resolutions, which facilitates the extraction of complex signal information. GoDec algorithm is used to difference the background and the anomalies. It determines MD in the end to identify the likely anomalies and map anomalies. Six hyperspectral datasets are experimented and it was established that WTHAD has a better performance in detection compared to the existing state-of-the-art hyperspectral anomaly detection techniques.

## Introduction

Hyperspectral imaging technologies allow the acquisition of three dimensional images that characterize spatial and spectral features of various ground objects. This technology provides spectral information by capturing hundreds of narrow and contiguous spectral bands. Such spectral properties can not only be used to provide an accurate discrimination of materials but also provide a large variety of more advanced applications of remote sensing. Of particular interest in this context is target detection which focuses on identifying meaningful objects in complex and diverse backgrounds. Hyperspectral target detection can be regarded as a binary classification problem, and an image is decomposed into two classes: targets regions and background. The general paradigms of detection strategies are placed broadly into two categories based on the availability of previous target data; supervised detection strategies that use known spectral signatures, and anomaly detection strategies that are required in situations where previous target data is not available^1,2^. This latter paradigm has identified hyperspectral anomaly detection as one of the key tasks, with the goal of determining pixels that do not follow the statistical characteristics of the background, and through which to find rare or unknown targets, with no reference to prior spectral knowledge^3^. As a result, numerous detection algorithms were created to provide a suitable model of background features. Such models then signal anomalies as pixels that significantly diverge the constructed background representation hence coming up with a separation between potential targets and the background. Yet in real life applications, the lighting, the surroundings, the sensor system, and neighboring terrestrial objects frequently influence the target spectrum determination. However, despite all these influences, the ability to automatically identify anomalous areas makes anomaly identification advantageous without prior information.

Global Reed Xiaoli (GRX) algorithm, a statistical based method, functions as a constant false alarm rate detector built upon generalized likelihood ratio test framework^4^. It operates under assumption that background follows a Gaussian distribution where probability density functions are estimated using mean vector and covariance matrix of background samples. GRX encounters difficulties with excessive false alarms in detection maps due to anomalies and noise embedded in background data. Local RX (LRX) is essentially a spatially adaptive variant of GRX statistic that substitutes a local normal model for global background model^5^. A double window is slid over each pixel in the image to find anomalies. It is expected that the inner window is the same extent as the feature of interest in the scene. Target pixels and background statistics are assumed to be able to be described in distinct subspaces employing Subspace RX (SSRX)^6^. The background characteristics are suppressed by projecting the background vector onto a subspace. Nevertheless, complex or nonlinear background distributions frequently present challenges for traditional RX like statistical detectors. Kernel RX (KRX) was created for such samples requiring more high level decision boundaries^7^. KRX employs nonlinear models. To translate input space into a multi-dimensional feature space, it uses kernel functions. In addition to KRX, researchers have proposed a series of enhancements within the RX framework, weighted RX, and linear filter based RX^8^. LSAD^9^ further improves LRX by incorporating a multi window strategy and subspace projection, while the robust nonlinear anomaly detection method addressed the issue of anomaly contamination with statistical modeling^10^. In this context, Target to Anomaly Conversion (TAC) can be used to provide a complementary view by reformulating the detection task in such a way that background statistics are less significant, and thus, the detection can be more reliable in the case where the target signatures are ambiguous or partial^11^.

Representation-based strategies address hyperspectral anomaly detection without relying on an explicit statistical background distribution. Instead, each pixel is expressed in the form of a linear mixture of atoms derived from a background dictionary, and anomalies are identified through the residuals of reconstruction. Within this category, collaborative representation, sparse representation, and low rank representation are commonly employed, with their distinction lying in the form of regularization imposed. The sparse representation based approach was initially developed by Chen et al.^10^ for hyperspectral target detection and later extended by Li et al.^12^ for anomaly detection tasks. SSEAD^13^ further advanced sparse representation by estimating the frequencies of dictionary atoms and applying enhancement strategies to refine anomaly scores. Li and Du^14^incorporated spatial information into CR, where anomalies were inferred from the reconstruction errors of surrounding pixels. Lin and Lin^15^ introduced super RPCA by embedding the collaborative representation detector (CRD) into robust principal component analysis (RPCA)^16^, effectively exploiting local information to achieve lower computational complexity. Lastly, Effective Anomaly Space (EAS) technique can inhibit the background factors by projecting the data to create an anomaly isolating subspace to achieve the ability to effectively separate the anomaly pixels out of the complicated hyperspectral backgrounds without previous target knowledge^17^.

Low Rank Representation (LRR), originally formulated by Liu et al.^18^ for subspace clustering, was later adapted for HAD and has been improved by subsequent studies. The low rank and sparse representation method^19^ leverages low rank nature of background dictionaries along with the sparsity characteristic of anomalies. To further suppress background effects, the effective anomaly space method^20^ was developed using independent component analysis and sparsity cardinality, enhancing the low rank and sparse representation based techniques. The low rank and sparse matrix decomposition (LRaSMD) method^21^ separates anomalies by decomposing hyperspectral data into low-rank, sparse, and noise parts via convex optimization. Expanding this framework, LSMAD^22^ applies the GoDec algorithm^23^ to extract low-rank and sparse components and employs MD differences for anomaly identification. Other works^24–26^ improve LRR by introducing more suitable constraints to increase accuracy. A novel anomaly detector by integrating the fractional Fourier transform (FrFT) with LRaSMD is introduced^27^. In comparison to the classical LRaSMD methods, which can be characterized by instability and lack of practicality, Feature Extraction and Background Purification Anomaly Detection (FEBPAD) uses a row-constrained LRaSMD (RC-LRaSMD) to provide a more robust separation. First, distinctive features of hyperspectral data are extracted via FrFT. Then, FEBPAD is utilized to effectively distinguish background from noise and anomalies. Beyond these approaches, other GoDec based and subspace recovery techniques have also been proposed. The hyperspectral anomaly detection method based on Laplacian matrix (HADLAP)^28^ differs from Euclidean distance–based detectors by employing MD to improve anomaly separation. Robust Subspace Recovery (RoSuRe) algorithm is also an extensively used algorithm in order to maximise the accuracy of data decomposition^29^, and later research improved the anomaly detection technique by adding MD to the sparse representation^22^. Building upon RoSuRe, the Sparse and Low Rank Matrix Decomposition (SLRMD) method^30^ extracts a sparse matrix from which an anomaly detector is constructed using MD. More recently, Hybrid Anomaly Detection Method (HADM)^31^ was proposed, in which the Laplacian matrix is incorporated into the anomaly detector having better detection results.

Over the last few years, hyperspectral anomaly detection has shown widespread use of deep learning (DL) strategies^32–34^. Models based on autoencoders (AE)^35^ are usually trained to detect networks by leveraging pixel level reconstruction errors. The robust graph autoencoder (RGAE)^36^ incorporates a graph regularization term derived from superpixel segmentation to maintain the spatial geometry of hyperspectral images. Auto-AD^37^ reconstructs the background using a fully convolutional AE enhanced with skip connections.Moreover, Pixel-associated Autoencoder (PAAE) enhances reconstruction with discrimination between normal and abnormal pixel in hyperspectral image by incorporating spatial related pixel data into the autoencoder architecture^38^. Compared to traditional autoencoder-based approaches, Guided Autoencoder (GAED) has a better capability of detecting anomalies because it incorporates a guiding mechanism to constrain the reconstruction process by using background priors^39^. The deep feature aggregation network (DFAN)^40^ introduces orthogonal spectral attention and background modeling, while FCAE-DCAC^41^ combines spatial–spectral attention with dual-clustering and adversarial consistency to improve AE-driven anomaly detection. Convolutional neural network (CNN)-based methods^42^ detect anomalies by training with both similar and dissimilar pixel pairs, measuring anomaly scores through average similarity with neighboring pixels. The spectral–spatial deep support vector data description algorithm^43^ applies a dual-stream deep convolutional AE framework to learn spectral spatial features. Song et al.^44^ used CNNs with LRR in order to increase the accuracy of detection. The low-rank embedding network^45^ jointly optimizes the AE and Gaussian mixture model (GMM) to increase adaptability. In addition, HADGAN^46^ integrates AE-based reconstruction constraints into generative adversarial networks (GANs), while a dual-GAN strategy^47^ has been proposed to more faithfully model the background distribution.

In addition to the previously discussed methods, other DL-based detectors have also been developed. GT-HAD^48^, for instance, leverages spectral spatial similarity to guide both anomaly and background reconstruction through a Transformer equipped with gating mechanisms. Similarly, the Transformer-based Autoencoder Framework (TAEF) significantly enhances the capability of explaining anomalies in complex hyperspectral scenes by modeling long-range spectral–spatial dependencies via self-attention mechanisms^49^. Moreover, background reconstruction based on a 3D Transformer Network (3DTR) enables more accurate background estimation and enhanced anomaly separability by jointly learning spectral and spatial correlations through a three-dimensional attention scheme^50^. A blind spot self supervised framework is introduced^51^. It predicts central pixel using its surroundings^52^, with subsequent studies^53,54^ refining the approach to better handle large-scale anomalies. The wavelet transform has been extensively used in the wavelet transform in many applications in order to perform trustworthy signal processing and analysis^55–57^. The wavelet based preprocessing is of great help to hyperspectral anomaly detection since with the help of discrete wavelet transform (DWT) the high-dimensional spectrum of each pixel is broken down into a collection of multiscale components^58–60^. The low frequency approximation coefficients still have the critical spectral signature of the scene, whereas the high frequency detail coefficients are mostly full of noise and redundant data. These detail coefficients are discarded or attenuated to make the transformed data more compact and less corrupted and this increases the contrast between anomalous targets and the surrounding background. This reduction in dimensionality and noise enhance detection accuracy and computation, making real time processing more feasible. Moreover, the multiresolution nature of wavelets enables the capture of both fine scale anomalies and broader spectral trends, providing a versatile foundation for robust hyperspectral anomaly detection techniques.

This study has been motivated by the fact that, although much has been done by statistical, representation-based, and deep learning-based hyperspectral anomaly detection systems, the separation of anomalies in the case of complex scenes in real world is a demanding issue. The current methods are typically vulnerable to background contamination, sensitivity to noise, or variable sparse decomposition especially when the background has sharp spectral variations or when the anomalies are weak, large scale, or spectrally correlated. Statistical detectors are also very sensitive to good background modeling assumptions and low rank and sparse decomposition methods might not be effective to isolate minute anomalies because of poor purification of the background. Despite the impressive performance of deep learning models, they are usually trained on large datasets, are expensive in terms of computing power and cannot be interpreted, which restricts their use in unsupervised or real-time applications. Due to these constraints, this paper suggests a coherent framework that uses reorganization of wavelet-domain features to enhance the correlation of the background, stabilize a low-rank decomposition, and enhance saliency of an anomaly after which strong statistical discrimination is used. The combination of the wavelet transform-based multiscale representation and the GoDec-based low-rank decomposition and modified MD analysis will enable the proposed method to obtain a stable, interpretable, and computationally efficient hyperspectral anomaly detection without any prior knowledge about the target. Overview of the proposed method and its key contributions can be outlined as follows:

1. A preprocessing stage is carried out using Haar WT, which reduces redundancy in hyperspectral data and enhances discriminative spectral-spatial features relevant to anomaly detection.
2. Subsequently, the GoDec algorithm is employed to decompose the transformed data into a low-rank background component and a sparse anomaly component, effectively isolating anomalies from the structured background information.
3. Finally, MD is performed on the low rank component for precise anomaly detection, exploiting its statistical capability to measure deviations from the background distribution.

WT is not used as a general preprocessing method but is used to reorganize the data into a feature space that explicitly allows low-rank and sparse decomposition. The spectral background components in space in the wavelet domain are more correlated and concentrated, which enhances the low-rank assumption in GoDec. On the contrary, anomalous pixels are likely to generate localized, discontinuous and high frequency responses that are more sparse and more salient with wavelet decomposition. This contrast enhances the ability to separate background and anomaly components as opposed to the original spectral space. In contrast to PCA where a global linear projection is applied and an anomaly signature can be contaminated within large components, WT offers a multi-scale and localized representation. This results in a more stable decomposition of GoDec and the following calculation of the MD receives the advantage of a denoised and structurally homogenized background model. The novelty of the study lies in the proposed WTHAD framework that integrates wavelet-domain feature extraction, low-rank decomposition, and statistical distance measurement into a unified anomaly detection model. The preprocessing of the wavelet is employed to enhance the background and anomaly separation as compared to other methods, and the MD evaluation is used to make the detection more accurate and robust. Consequently, WTHAD has a better detection performance and lower false alarm rates with respect to conventional methods.

The structure of this paper is as follows. Section “Introduction” gives a brief introduction and reviews the existing literature on hyperspectral anomaly detection. Section "Material and method" presents a detailed description of the proposed method, along with the description of the hyperspectral datasets used in the experiments. Section “Experimental results” presents experiment results including quantitative assessments and comparisons. Section “Discussion” discusses analysis and interpretation of the results. Finally, Section “Conclusions” summarizes the conclusions of the study.

## Material and method

In the next section, the proposed framework of the anomaly detection will be described and the hyperspectral datasets on which it is to be tested will be introduced. First, an overview of the proposed method is provided, with a focus on its key elements and the step-by-step procedure. Then, the details of the real-world hyperspectral datasets in 1 are employed in the experiments.

### Proposed method

Wavelet transform can analyze a signal at varying frequencies with varying resolutions, and thus can be able to capture complex signal characteristics. The primary idea is to model the original data with the scale feature and separate the low-frequency part, which represents the overall characteristics of the signal. An expansion in the scale also decreases the suitability of the function of the scale to characterize the initial signal, and thus it expands the deviation. Accordingly, the wavelet function is introduced to model this difference. The high-frequency part of the data which holds detailed information is captured in the function. Specifically, WT decomposes the original data in the form of approximation coefficients and detail coefficients under the action of the scale function and the wavelet function^61,62^. HSI data cube is presented as $X \in \mathbb {R}^{M \times N \times L},$ where *M* and *N* correspond to the spatial dimensions, and *L* indicates the number of spectral bands. For each pixel (*i*, *j*), its spectral signature is extracted as $x_{i,j} = \bigl [ X(i,j,1), X(i,j,2), \dots , X(i,j,L) \bigr ] \in \mathbb {R}^L .$ Given the data as represented through the scale function $\beta = \{\beta _1, \beta _2, \ldots , \beta _{N-1}\}$, and the wavelet function $\varepsilon = \{\varepsilon _1, \varepsilon _2, \ldots , \varepsilon _{N-1}\}$, by projecting the data *X* onto $\beta$ and $\varepsilon$, the corresponding approximation coefficients are obtained. Haar WT yields its approximate and detailed outputs through

1 $$\begin{aligned} {\left\{ \begin{array}{ll} a_{j,k} = \sum \limits _{i} f(i - 2k) a_{j-1,i}, \\ d_{j,k} = \sum \limits _{i} h(i - 2k) a_{j-1,i}, \end{array}\right. } \end{aligned}$$

where *j* and *k* correspond to the scaling and translation parameters; $a_{j,k}$ is the approximation coefficient, and $d_{j,k}$ is the detail coefficient. Transformed feature vector is obtained by concatenating the approximation as in $y_{i,j} = \bigl [ a_1, a_2, \dots , a_{L/2}, \; d_1, d_2, \dots , d_{L/2} \bigr ].$

Finally, the wavelet features are truncated to the original length *L* and their absolute values are taken as in $d_{i,j} = \bigl | \textbf{y}_{i,j}(1:L) \bigr | .$ Thus, the feature–extracted HSI data cube is given by $\textbf{D}(i,j,:) = \textbf{d}_{i,j}, \quad \textbf{D} \in \mathbb {R}^{M \times N \times L}.$ After applying the wavelet transform, the data $D\in \mathbb {R}^{M \times N \times L}$ can be represented as $D=[D_1, D_2,... , D_L], D\in \mathbb {R}^{p\times L}$ where *p* indicates the total number of pixels. This formulation corresponds to objectives of hyperspectral anomaly detection employing low-rank and sparse matrix decomposition. Accordingly, the model of *D* obtained by wavelet transform can be expressed by addition of background matrix *B* and sparse matrix *S* as $D = B + S.$ The background in hyperspectral images is typically redundant and can be efficiently modeled within a low-dimensional subspace, since anomaly pixels occupy only a very small proportion. Exploiting the mentioned characteristics, the reshaped HSI consists of a low-rank matrix B representing the background and a sparse matrix S representing the anomalies. Consequently, hyperspectral anomaly detection is expressed as the following minimization problem:

2 $$\begin{aligned} \min _{B, S} {\Vert D - B - S \Vert }^2_F, rk(B)\le r, \Vert S\Vert _0\le kN \end{aligned}$$

here, r and k denote the upper limits of the rank of matrix B and the cardinality of S, respectively. Although the minimization task is solved by employing the alternating direction method of multipliers (ADMM), this approach becomes computationally expensive due to the reliance on singular value decomposition (SVD). To alleviate this issue, the GoDec algorithm, summarized in the algorithm 1 is employed. GoDec accelerates the process by performing a fast low-rank approximation through bilateral random projections (BRP)^63^. The optimal solution is acquired by iteratively computing matrices *B* and *S* in GoDec algorithm.

**Algorithm 1:**
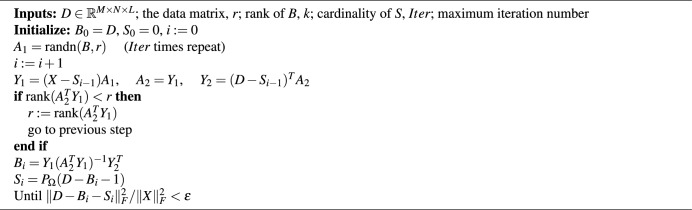
GoDec algorithm^23^.

However, nonzero elements in matrix *S* may appear at arbitrary locations when using GoDec. This behavior is suitable for foreground moving object detection but is not ideal for hyperspectral anomaly detection. Matrix *S* exhibits a row-sparsity property because each row is associated with the anomaly probability of a particular pixel. In this work, a new approach is proposed to construct the subset for forming *S*. In addition to enforcing the sparsity property, the subset is stabilized by fixing it prior to applying decomposition into low-rank and sparse components. Specifically, the initial results are obtained from WT-MD. WT-MD is used before GoDec to estimate an initial image of the likelihood of an anomaly, relative to which the pixels in S are grouped into a set of candidates, permitted to assume nonzero values. This evenly balances the sparsity, bringing out significant anomalies, and inhibiting entries due to noise, without interfering with the low-rank background B.

A detailed explanation of the WT-MD stage is provided as follows. Let the wavelet-domain hyperspectral data be rearranged as $X \in \mathbb {R}^{MN \times K}$, where each row $x_i \in \mathbb {R}^{K}$ corresponds to the spectral feature vector of the $i$-th pixel. The wavelet-domain representation is obtained as

3 $$\begin{aligned} \textbf{Z} = \mathscr {W}_{\text {Haar}}(\textbf{X}), \end{aligned}$$

where $\mathscr {W}_{\text {Haar}}(\cdot )$ denotes the one-dimensional Haar wavelet transform applied independently to each pixel along the spectral dimension. An initial anomaly likelihood is computed for each pixel using a MD-based detector. Unlike the classical RX detector, where background statistics are estimated directly from the observed data, the WT–MD step estimates the background distribution from the low-rank background B. The background mean vector $\mu \in \mathbb {R}^{K}$ and covariance matrix $\Sigma \in \mathbb {R}^{K \times K}$ are estimated from the data as $\mu = \frac{1}{MN} \sum B$ and $\Sigma = \frac{1}{MN} \sum (B - \mu )(B - \mu )^{\top }$. The WT-MD score for each pixel is then given by modified MD as follows:

4 $$\begin{aligned} MD_{WT} = (Z - \mu )^{\top } \Sigma ^{-1} (Z- \mu ). \end{aligned}$$

The resulting scores $dist_i$ are used to define a fixed candidate support set $\Omega = \left\{ i \mid MD_{WT_i} \in \textrm{Top}\text {-}k(\{ MD_{WT_j} \}) \right\}$, where only the $k$ pixels with the highest WT-MD scores are retained. The support set is expanded along the spectral dimension to form a binary mask $\Omega \in \{0,1\}^{MN \times K}$. At this stage, the sparse component is initialized as S = 0, and the low-rank component is initialized as B = X. By fixing the support set $\Omega$ prior to any iterative optimization, the sparsity structure is explicitly constrained, ensuring row-wise sparsity consistency and preventing arbitrary sparse activations caused by noise.

Within the proposed framework Haar wavelet transform is applied on initial hyperspectral data after which all the subsequent processing is carried out in the wavelet domain. The WT step is utilized on the wavelet-transformed data before it undergoes low-rank and sparse decomposition, only to identify a fixed candidate sparse support. To ensure full reproducibility, all wavelet-related parameters are explicitly fixed in this work. A single-level one-dimensional Haar wavelet transform is applied independently to each pixel along the spectral dimension. The Haar low-pass and high-pass filters are defined as $f = \left[ 1/\sqrt{2},\; 1/\sqrt{2} \right]$ and $h = \left[ 1/\sqrt{2},\; -1/\sqrt{2} \right]$ and calculated by the following equations as a part of the code of the proposed method.

5 $$\begin{aligned} \textrm{approx}(k) = \frac{x(2k-1) + x(2k)}{\sqrt{2}}, \textrm{detail}(k) = \frac{x(2k-1) - x(2k)}{\sqrt{2}} \end{aligned}$$

In order to explain the significance of the last detection step, the background matrix B that has been obtained by GoDec based on low-rank background is considered a dominant background subspace as opposed to a perfectly purified background model. Whereas strong and high energy anomalies can be much more segregated into the sparse component S, weak, spatially spread, or spectrally correlated anomalies can still be only partially segregated in B by the approximate nature of low-rank sparse decomposition. Using modified MD hence is a subspace-consistency test showing pixels that are statistically inconsistent with the prevailing background structure even when their residual energy is not high enough to stand out in S. Accordingly, the initial result obtained from the WT-MD based detector is considered as anomaly likelihood for each pixel, which is exploited for background construction. Finally, it is employed in modified MD as calculated in $MD_{WT}$ for error elimination and anomaly detection.

6 $$\begin{aligned} MD_{WT}(Z)=(Z-{\mu })^T\Gamma ^{-1}(Z-{\mu }) \end{aligned}$$

where $\mu$ and $\Gamma$ are mean and covariance matrices obtained with $md_{wt}$ by the equation 4. Finally, anomaly detection maps are obtained.

The proposed algorithm combines wavelet-domain feature extraction, low-rank and sparse decomposition, and modified MD in one hyperspectral anomaly detection system. The algorithm framework is represented in Algorithm 2.

**Algorithm 2:**
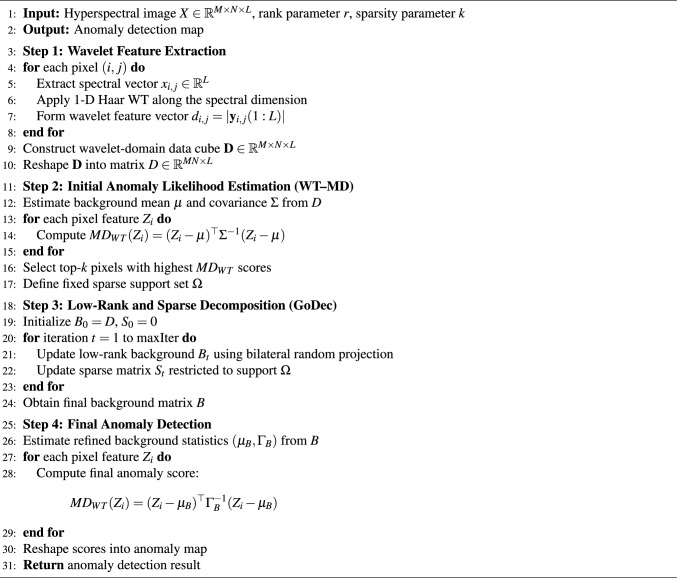
Proposed WT-GoDec-MD framework.

### Hyperspectral datasets

**Fig. 1:**
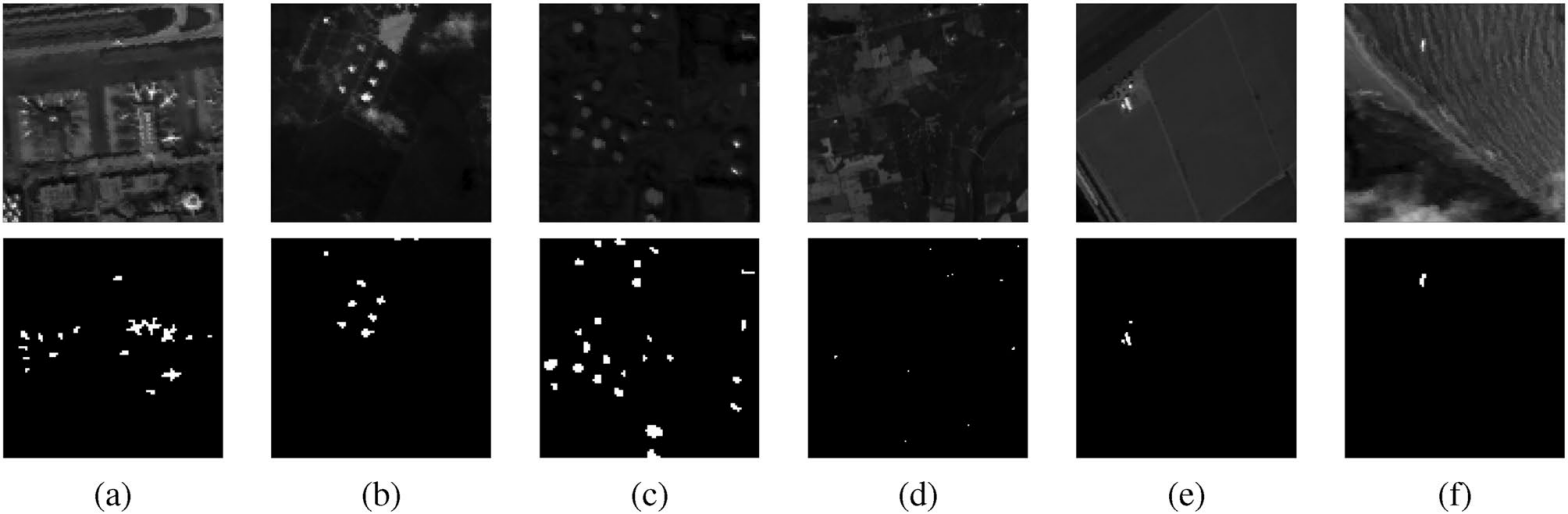
False color and ground truth images. **a**) ABU Airport 3. **b**) ABU Urban 1. **c**) ABU Urban 4. **d**) Hyperion. and **e**) Salinas. **f**) ABU Beach 3.

In this section, experiments are conducted using six publicly available HSI datasets^64^. The details of the images are provided in the Table 1. Figure 1 presents the original band images alongside the corresponding ground truth images.

**Table 1:** Information of hyperspectral datasets.

Datasets	Spatial Size	Bands	Resolution	Location	Sensor	Anomaly Type	Anomaly
**ABU Airport 3**	$100\times 100$	205	7.1m	Los Angeles	AVIRIS	Plane	170
**ABU Urban 1**	$100\times 100$	204	17.2m	Texas Coast	AVIRIS	Buildings	67
**ABU Urban 4**	$100\times 100$	205	7.1m	Los Angeles	AVIRIS	Buildings	272
**Hyperion**	$150\times 150$	155	10m	Indiana	Hyperion	Small objects	17
**Salinas**	$150\times 200$	204	3.7m	Salinas Valley	AVIRIS	Field	42
**ABU Beach 3**	$100\times 100$	188	4.4m	Bay Champagne	AVIRIS	Field	11

Airport Beach Urban (ABU) Dataset^65^: Figure 1 (a), (b), (c), (f) display the sample images and the associated reference detection maps from the ABU dataset, with anomalous targets occupying 170, 67, 274, and 11 pixels, respectively. The sample images, each of size $100 \times 100$ pixels, were manually cropped from larger images acquired from the Airborne Visible/Infrared Imaging Spectrometer (AVIRIS) dataset. Some characteristics of these images are summarized in the Table 1. As indicated in the table most of the images were captured by the AVIRIS sensor, except for Fig. 1 (d) and (e).

Hyperion^66^: The dataset was acquired in 2008 over an agricultural region in Indiana, USA, using Hyperion imaging sensors. In this dataset, small objects such as silos and rooftops that blend into the surrounding environment are considered anomalies. There are 17 pixels in total. The dataset contains 155 spectral bands with spatial dimensions of $150 \times 150$ pixels.

Salinas^64^: The hyperspectral scene used in the experiments was collected by the AVIRIS sensor over an agricultural test site in Salinas Valley, California. The scene comprises 150 lines by 200 samples, with 204 spectral bands remaining after the water absorption and noisy bands have been removed. Anomaly objects have a total of 42 pixels in the data.

## Experimental results

**Fig. 2:**
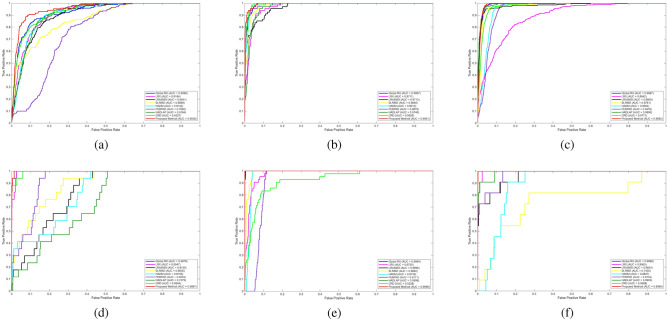
ROC curves of the proposed method (WTHAD) and comparison methods on six public HSI datasets: **a**) ABU Airport 3. **b**) ABU Urban 1. **c**) ABU Urban 4. **d**) Hyperion. and **e**) Salinas. **f**) ABU Beach 3.

**Fig. 3:**
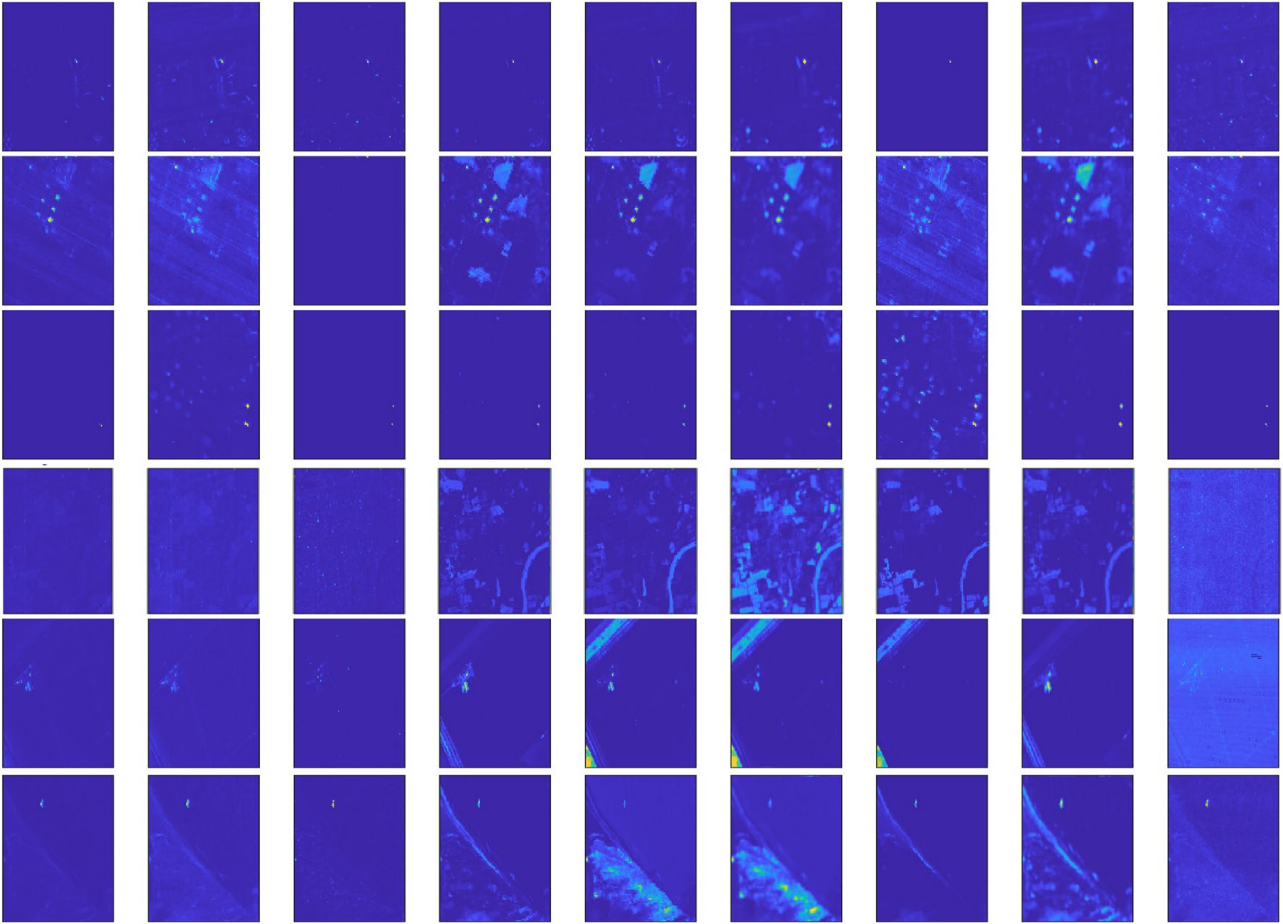
Visual comparison of anomaly detection maps for six public hyperspectral datasets. From top to bottom: ABU Airport 3, ABU Urban 1, ABU Urban 4, Hyperion, Salinas, and ABU Beach 3. From left to right: Proposed, GRX, LRX, LRASMD, SLRMD, HADM, FEBPAD, HADLAP, and CRD.

In this section, the proposed method, referred to as WTHAD, is evaluated against eight state-of-the-art hyperspectral anomaly detection approaches: GRX^4^, LRX^5^, LRASMD^21^, SLRMD^30^, HADM^31^, FEBPAD^27^, HADLAP^28^, and CRD^16^. These methods are applied to six HSI datasets. To compare the methods, ROC (Receiver Operating Characteristics) curves are plotted, and AUC (Area Under ROC Curve) values are calculated. These two metrics are mostly used techniques for the evaluation of anomaly detectors in the literature. ROC curves shown in Fig. 2 illustrates the performance comparison of the anomaly detection methods. The x-axis represents the false alarm rate on a logarithmic scale, while the y-axis indicates the detection probability. AUC values are represented in the Table 2. In addition to ROC and AUC analysis, two-dimensional results for each methods’ anomaly maps are presented in Fig. 3 to visualize the results as an image.

Figure 2 (a) shows comparison of different hyperspectral anomaly detection algorithms based on their performance in detection The proposed method, dubbed as WTHAD, is obviously superior in performance when compared to the rest of the methods as it has the highest AUC of 0.9538 which depicts the best ability in distinguishing anomalies and the background at varying false positive rates. The case with traditional methods, like GRX (0.9288) and LRX (0.9184), is competitive but significantly less productive, which means that they are more likely to raise false alarms or fail to detect. The methods that are based on matrix decomposition, such as LRSAMD (0.9041) and SLRMD (0.8689) are not very bad but still inferior to the provided method. On the same note, subspace and dictionary learning-based methods including HADM (0.9142), FEBPAD (0.7692) and HADLAP (0.9164) are also quite decent in terms of the detection functionality; however, they still are not as effective as the proposed solution. CRD method (0.9327) is also performing well and is also the nearest rival; it is still low compared to the offered technique. Comprehensively, the findings demonstrate the strength and efficiency of WTHAD which has a consistently better trade-off between the sensitivity and specificity of detection, which makes it exceptionally applicable in real-life hyperspectral anomaly detection.

Figure 2 (b) shows a comparative evaluation of state-of-the-art anomaly detection algorithms on hyperspectral data for ABU Urban 1 dataset. The proposed method, red curve, achieves the best detection performance with an AUC of 0.9651, indicating its superior capability in balancing true positive rate and false positive rate. Classical detectors such as GRX and LRX show acceptable results; 0.9907 and 0.9711 respectively, yet they are outperformed by more advanced methods. Low rank and sparse matrix representation-based techniques, including LRASMD (0.9712) and SLRMD (0.9848), achieve competitive accuracy but still remain slightly below the proposed approach. Hybrid and subspace-based methods, such as HADM with 0.9913 AUC, FEBPAD with 0.9876 AUC, and HADLAP with 0.9740 AUC, provide reliable performance, with HADM being particularly strong, though not surpassing the proposed model. Meanwhile, the CRD method with 0.9920 AUC is the closest competitor, yet it still falls marginally short of the proposed technique. Overall, these findings point to the fact that the proposed method is the most resistant to anomaly detection, showing greater reliability with a large spectrum of false positive rates and proving its superiority to the classical and modern competitive approaches.

Figure 2 (c) illustrates the detection performance for ABU Urban 4 dataset. WTHAD is maximally accurate at 0.9892, and it clearly indicates that WTHAD is highly effective at detecting abnormalities versus the background with the various false positive rates. The performance of traditional algorithms LRX (0.8842) is comparatively worse hence depicting their flaws in sophisticated cases of detection. More developed methods that are founded on low-rank and sparse modeling, including LRASMD (0.9808), HADLAP (0.9890) have a better accuracy, with HADLAP becoming one of the most serious rivals. Subspace and representation based approaches such as HADM (0.9564), FEBPAD (0.9476) and SLRMD (0.9761) are effective and indicate the usefulness of structural modeling in the detection of anomalies. CRD (0.9771) is an average in terms of performance, being better than other methods, but behind others. On balance, the findings indicate that the suggested approach has the highest detection potential, which is significantly better than the classical and modern-day competitors and guarantees the presence of strong anomaly detection in hyperspectral imaging.

Figure 2 (d) shows the results for Hyperion dataset. GRX method attains an AUC of 0.9978, and LRX also gives an AUC of 0.9947. Conversely, LRASMD and SLRMD do not achieve such high results as they have AUC of 0.8155 and 0.883 in, respectively. The results of HADM and FEBPAD are moderate (UC of 0.8159 and 0.9204), and HADLAP performances are lower (UC of 0.7275). CRD method on the other hand gives a significantly better result with a AUC of 0.9944. Lastly, the proposed method achieves the highest AUC of 0.9987 which proves its superiority in the task of detecting anomalies making it superior to all other methods. The results clearly indicate that the suggested technique offers trustworthy performance over a large scale of false positive rates with respect to the available techniques.

Figure 2 (e) illustrates the ROC curves derived from the Salinas dataset for an in depth assessment of hyperspectral anomaly detection techniques, along with their corresponding AUC values.GRX technique offers the near perfect detection capacity with AUC of 0.9994 with LRASMD algorithm slightly outperforming it with 0.9996. Conversely, the LRX approach yields less AUC of 0.9755 which demonstrates the weaknesses of the approach to characterize the minor abnormalities in the intricate spectral-spatial distribution of the Salinas scene. Equally, SLRMD method is superior to LRX in terms of the AUC of 0.9882, yet, it is still inferior to HADLAP, LRASMD, and GRX, which indicates that its residual modeling dependence does not generalize as well. The HADM approach acheives competitive AUC of 0.9739, whereas FEBPAD approach measures 0.9171 which still depict moderate performance but will be prone to false positives in some areas of the ROC curve. Conversely, the CRD technique has the lowest score among them with an AUC of 0.9228 only, which validates its low-abilities to deal with the spectral complexity of the Salinas data. HADLAP being the strategy with the strongest background-adaptive approach has an AUC of 0.9998 which is more robust than the other background-adaptive strategies. Lastly, the proposed approach, which is denoted by the red curve, obtains the highest AUC of 0.9998 and then HADLAP, making it better than all the current techniques in the whole range of false positive rate. Beside proving the high accuracy and strength of the proposed approach, this finding also reveals the capability to preserve the performance of the anomaly detection in the case of the Salinas dataset as the conditions are rather demanding.

Figure 2 (f) presents the ROC curves derived from the ABU Beach 3 dataset. The proposed method attains achieving the highest performance with an AUC of 0.9999, closely followed by GRX and CRD at 0.9998. Among residual modeling models, LRX has a moderate AUC of 0.9965, as compared to SLRMD which has a much lower AUC of 0.7430, indicating very strong sensitivity to false positives despite low false positive rates. Background-adaptive and projection versions produce better results: HADLAP makes 0.9903, FEBPAD makes 0.9734 and HADM makes 0.8827. As a whole, the ranking throughout the whole false positive rate spectrum is led by the proposed method, then by the RX-based baselines, with residual strategies being the last ones as they help highlight the effectiveness and discriminative capability of proposed detector on the image of ABU Beach 3.

**Table 2:** AUC values.

Method	ABU Airport 3	ABU Urban 1	ABU Urban 4	Hyperion	Salinas	ABU Beach 3
Proposed (WTHAD)	0.9538	0.9951	0.9892	0.9987	0.9998	0.9999
GRX	0.9288	0.9907	0.9887	0.9978	0.9994	0.9998
LRX	0.9184	0.9711	0.8842	0.9947	0.9755	0.9965
LRASMD	0.9041	0.9712	0.9808	0.8155	0.9996	0.9624
SLRMD	0.8689	0.9848	0.9761	0.8830	0.9882	0.7430
HADM	0.9142	0.9913	0.9564	0.8159	0.9739	0.8827
FEBPAD	0.7692	0.9876	0.9476	0.9204	0.9171	0.9734
HADLAP	0.9164	0.9740	0.9890	0.7275	0.9998	0.9903
CRD	0.9327	0.9920	0.9771	0.9944	0.9228	0.9998

Table 2 presents a detailed comparison of the values of the AUC on six hyperspectral samples, and the proposed method is superior. In the case of ABU Airport 3 scene, the given approach gives a higher AUC of 0.9538 than CRD (0.9327), GRX (0.9288), LRX (0.9184), and HADM (0.9142), with the lowest value of FEBPAD standing at 0.7692, which demonstrates the low robustness. In the case of the ABU Urban 1 data, the suggested method has the advantage 0.9951, which is larger than GRX (0.9907), HADM (0.9913) and FEBPAD (0.9876). In ABU Urban 4, the results of the proposed approach (0.9892) and HADLAP (0.9890) are almost similar yet LRX performs much worse with 0.8842, which demonstrates the ineffectiveness of residual-based modeling in urban complex setting. A more vivid difference could be noticed in the Hyperion dataset: the offered approach shows 0.9987, which is obviously higher than LRASMD (0.8155), SLRMD (0.8830), and HADM (0.8159), and GRX (0.9978) and LRX (0.9947) are also competitive but somewhat lower. Salinas data also proves the strength of the proposed method with the highest score of 0.9998, equal to that of HADLAP, and by far larger than FEBPAD (0.9171) and CRD (0.9228). Lastly, the proposed approach yields an almost optimal AUC of 0.9999 in ABU Beach 3 data slightly better than GRX and CRD (0.9998 and 0.7430, respectively). Collectively, these findings are consistent and confirm that the proposed approach provides more stability and accuracy in a variety of scenes, being more effective than RX-based detectors or background-adaptive or residual modeling-based methods, and makes it one of the optimal solutions to hyperspectral anomaly detection.

Figure 3 shows the 2D detection maps of the proposed approach and the competing algorithms on six publicly available HSI datasets. It would be noted that the suggested approach yields clearer and smaller anomaly maps with targets clearly defined and background areas suppressed well. RX-based methods, GRX and LRX, on the other hand, tend to add background clutter, whereas residual modeling methods, including LRASMD and SLRMD, are likely to produce scattered false alarms, especially in dense urban and vegetation scenes. Background adaptive procedures like HADM, FEBPAD, and HADLAP have better performance compared to residual based methods, but still have poor consistency in damping out the heterogeneous background structures. Comprehensively, the suggested approach is the most visually consistent and robust one, and it generates the well-localized areas of anomaly with the minimum amount of noise, which proves its suitability concerning the real hyperspectral anomaly detection tasks.

**Fig. 4:**
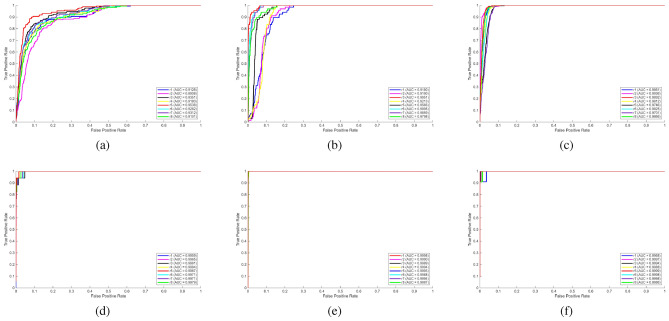
Parameter r (rank) analyses **a**) ABU Airport 3. **b**) ABU Urban 1. **c**) ABU Urban 4. **d**) Hyperion. **e**) Salinas. **f**) ABU Beach 3.

Figure 4 provides a comparative study of performance at various rank (r) values. As observed in the curves, the selection of ranking plays a huge role in the detection performance. As seen from the curves, rank selection has a significant effect on detection performance. In particular for Airport 3, the r=5 value provides the best overall performance with the highest AUC at 0.9538, while it is observed that AUC decreases relatively at very low or very high rank values. The highest AUC value was obtained for the Hyperion dataset r=5 with an AUC 0.9987, indicating that this rank value provides the best detection performance of the method. The highest detection performance was also achieved for r=5 for Beach 3 with an AUC 0.9999, yielded the best result compared to other rank values. As for Salinas dataset, the highest AUC value was obtained for r=1 with an AUC of 0.9998, indicating that this rank value offers the best detection performance of the method. The most successful result for Urban 4 dataset was obtained for r=3 with an AUC of 0.9892 value indicates that the method offers superior discrimination performance under this parameter compared to other rank options. In the case of Urban 1 data, the highest results of the proposed method are achieved at rank r = 3 with an AUC of 0.9951, indicating the highest value over the other rank values. Overall, the rank parameter was changed in the range of 1 to 8 in order to examine its impact on detection performance in various datasets. The findings indicate that moderate rank values tend to be better than very low or very high rank values, indicating the need to focus on the selection of the rank.

**Table 3:** Runtime results in seconds.

Method	ABU Airport 3	ABU Urban 1	ABU Urban 4	Hyperion	Salinas	ABU Beach 3
Proposed (WTHAD)	2.4787	2.5452	2.4842	4.1009	6.8881	2.2531
GRX	0.10013	0.097655	0.10974	0.16804	0.28097	0.089706
LRX	13.578	14.164	13.618	19.107	40.195	12.031
LRASMD	0.038153	0.027992	0.028852	0.044042	0.070142	0.026908
SLRMD	0.13484	0.11469	0.12558	0.18569	0.31706	0.10956
HADM	1.8878	1.9121	2.0546	1.7093	5.2982	1.4169
FEBPAD	2.7112	2.7668	2.7056	4.4051	7.4474	2.4898
HADLAP	1.8389	2.4526	2.0269	1.6758	5.025	1.3139
CRD	8.9158	9.9685	9.073	16.095	28.602	8.6979

The computational time (in seconds) of the proposed WTHAD method and the comparative algorithm on six hyperspectral datasets have been reported in Table 3. The findings denote that WTHAD gives a positive balance between detection performance and computational efficiency. Whereas, it is true that simple statistical detectors like GRX and LRASMD have shortest running times, this is at the expense of a limited model ability. In comparison, WTHAD often takes much less time than less computationally intensive algorithms like LRX and CRD on all datasets, and equal or better time on FEBPAD than FEBPAD. In addition, the scalability behavior of the proposed method is stable, and the runtime increases moderately in large-scale datasets, including Hyperion and Salinas. These findings indicate that WTHAD offers a viable tradeoff between accuracy and efficiency thus it would be appropriate when it comes to hyperspectral anomaly detection in real-life situations.

**Fig. 5:**
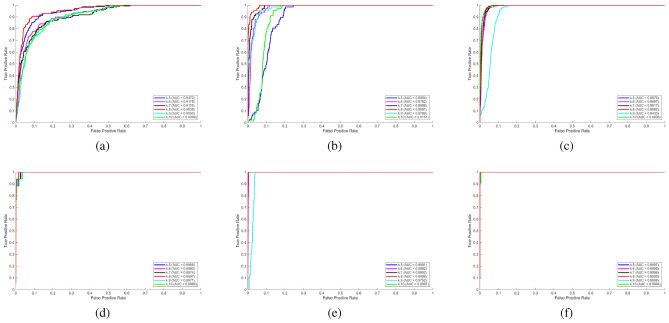
Parameter k analyses **a**) ABU Airport 3. **b**) ABU Urban 1. **c**) ABU Urban 4. **d**) Hyperion. and **e**) Salinas. **f**) ABU Beach 3.

Figure 5 shows the analysis of the cardinality parameter k. When all dataset results are considered together, proposed method has the highest AUC values with k value $0.8\times M \times N$. As seen from the figures that the ROC curves tend to converge towards the upper left corner at a faster rate. Thus, the highest detection performance is reached. It indicates that this value of k is a balanced and stable parameter choice across scenes and data sets.

### Ablation study

An ablation study was performed to assess the contribution level of each of the modules within the proposed framework by eliminating the WT, GoDec-based low-rank decomposition, and MD sequentially. As shown in Table 4, the entire model is the most successful in terms of detection performance on all benchmark datasets, proving the complementary aspects between the offered modules. The elimination of WT module causes a visible deterioration of the results, especially on ABU Airport 3 and ABU Urban 1, which magnifies the significance of multi-scale spectral representation and spatial representation. The removal of the GoDec module also causes performance to degrade, with the largest decrease in ABU Beach 3, and so it is clear that the low-rank background modeling is important in reducing structured clutter and increasing the separability of anomalies.

**Table 4:** Ablation study.

Method	ABU Airport 3	ABU Urban 1	ABU Urban 4	Hyperion	Salinas	ABU Beach 3
Proposed method	0.9538	0.9951	0.9892	0.9987	0.9998	0.9999
without Wavelet	0.9277	0.9732	0.9846	0.9949	0.9991	0.9996
without GoDEC	0.9366	0.9864	0.9872	0.9978	0.9994	0.9570
without MD	0.9135	0.9847	0.9822	0.9973	0.9991	0.9995

Secondly, substituting the MD-based detection step leads to further performance loss and particularly on ABU Airport 3, which proves to be effective in the modeling of the background statistics. In general, these findings support the assumption that every module is important to the ultimate accuracy of detection, and their combination provide a strong and better framework of anomaly detection.

## Discussion

Figure 2 and Table 2 that were obtained in the experiment illustrate the overall performance of the proposed WTHAD method. The significant attributes to the action include the wavelet-based preprocessing, the low-rank separation of the background by GoDec, and the last scoring based on MD. The overwhelming performance margin is seen in the Hyperion dataset. WTHAD has high performance on the Hyperion dataset where WTHAD has 0.9987 AUC, whereas LRASMD and SLRMD reduce to 0.8155 and 0.8830, respectively. This gap can be attributed to the images of Hyperion nature: they have powerful sensor specific noise, high dimensionality of the spectral, and non-uniform background structure. Low-rank residual based models, in contrast, are based on residual forms of the low-rank model. LRASMD and SLRMD are very sensitive to noise contamination and this is the reason why they are not effective in this environment. On the Salinas dataset, where there is strong spectral variability and dense vegetation backgrounds. WTHAD once again shows almost ideal performance (0.9998). Conversely, such datasets as ABU Beach 3 expose situations where conventional detectors still exist competitive. GRX and CRD are within the AUC at 0.9998 which is very similar to the 0.9999 of WTHAD. This is expected because the ABU Beach 3 has rather homogeneous regions in the background which include the classical covariance based statistics (GRX) and residual modeling (CRD) are already very effective. WTHAD is, nevertheless, the best in such simpler scenes, though it can provide only marginal gains, and stressing that it has the greatest benefits in complicated or noisy settings. Across the urban datasets, ABU Airport 3, ABU Urban 1, and ABU Urban 4, WTHAD has a steady improvement compared to LRASMD and SLRMD. The urban imagery is usually heterogeneous and mixed pixels, which are the conditions when the wavelet-based is applied. The anomaly signatures and the stabilization of the RX based detectors also do fairly well but fail to make complete use of the multi resolution spectral information and result in small but consistent. When the spectral composition is large or sensor noise or other complex background clutter is an issue, an important use of wavelet analysis is available advantage. Even in plain scenes having flat and similar backgrounds, WTHAD is equally matched or marginally outperforms classical detectors, having a strong performance with no loss of generality. This mutuality of strength is indicated by this data dependent behavior of the components of the method and discusses its outstanding and consistent performance on a variety of hyperspectral situations.

According to the findings represented in Figs. 4 and 5, the proposed method shows high and stable performance in detecting anomalies on various datasets under the condition of the right choice of the parameter settings. The rank parameter analysis indicates that the ability to detect is very sensitive to the selection rank with the highest AUC values usually obtained at moderate rank values but not at either very high or very low rank values. The best ranks are consistently between 3 to 5 and this indicates that there is a good balance between the flexibility and stability of the model. In addition, it is seen that the highest detection performance is obtained at rank values of 5 with AUC values 0.9538, 0.9999, and 0.9987; rank 1 with 0.9998 AUC and rank 3 with AUC 0.9951 and 0.9892. They are about 99% in overall. These findings show that appropriate rank selection plays an important role in maximizing the performance of the method. Equally, the cardinality parameter analysis shows that setting $k = 0.8\times MN$ produces the best overall AUC values of all datasets, and the ROC curves approach the upper left corner faster. While the k value is lower or higher than this value AUC value decreases which is also deceasing the detection performance. The parameter setting provides a stable and well balanced configuration, which allows to achieve higher performance in detecting anomalies in a variety of scenes and hyperspectral data.

## Conclusions

This study introduces a new approach for detecting anomalies in hyperspectral images and evaluates its performance on six benchmark datasets. Proposed WTHAD framework integrates WT with GoDec and MD to enhance hyperspectral anomaly detection. By decomposing each pixel spectrum into low-frequency approximation coefficients and high-frequency detail coefficients, WT emphasizes dominant spectral features while reducing noise. The GoDec decomposition is then used to part the low rank background out of the sparse anomaly structures to allow further refined localization of the anomalies. The experimental data show that this technique has the highest ROC and AUC statistics than the state of the art techniques, including GRX, LRX, LRASMD, SLRMD, HADM, FEBPAD, HADLAP, and CRD. These strengths notwithstanding, there are still computational efficiency issues, as well as problems with manipulating highly heterogeneous scenarios. Future directions may include light weight implementations of WT and GoDec, using semi-supervised approaches and use of prior knowledge to minimize false alarms and permit the real time applicability of such strategies in complex remote sensing systems.

## Data Availability

The datasets generated and/or analysed during the current study are available at, ABU and Salinas Datasets and Hyperion dataset.

## Code availability

The code developed and utilized in this study were uploaded to Zenodo repository with the DOI 10.5281/zenodo.18671521.
